# Homeostatic plasticity in patients with disorders of consciousness detected by combined stimulation: a study protocol

**DOI:** 10.3389/fneur.2025.1503946

**Published:** 2025-03-11

**Authors:** Jingwen Wang, Fangfang Shou, Qiuyi Yu, Xulan Lu, Yuwen Wan, Wangshan Huang, Nantu Hu, Zhenyi Jin, Xinru Shan, Steven Laureys, Haibo Di

**Affiliations:** ^1^International Unresponsive Wakefulness Syndrome and Consciousness Science Institute, School of Basic Medicine, Hangzhou Normal University, Hangzhou, China; ^2^College of Life and Environmental Sciences, Hangzhou Normal University, Hangzhou, China; ^3^Jing Hengyi School of Education, Hangzhou Normal University, Hangzhou, China; ^4^Canada Excellence Research Chair in Neuroplasticity, CERVO Brain Centre, Laval University, Quebec, QC, Canada

**Keywords:** transcranial direct current stimulation, transcranial magnetic stimulation, preconditioning, non-invasive transcranial brain stimulation, treatment, metaplasticity

## Abstract

**Background:**

Non-invasive neuromodulation (NIN) techniques have been widely utilized in treating patients with disorders of consciousness (DoC), but their therapeutic effects have been inconsistent. Given the reliance of NIN techniques on synaptic plasticity, and the potential impairment of synaptic plasticity (particularly homeostatic plasticity) resulting from severe brain injury, it is possible that the variation in therapeutic effects is due to alterations in homeostatic plasticity in patients with DoC. Therefore, this study will use preconditioning TMS to examine the retention of homeostatic plasticity in patients with DoC.

**Methods:**

We will enroll 30 patients with DoC and 15 healthy controls and randomize the order of their sessions. According to the priming protocol, the trial was divided into three different sessions with a 2-day break between each session. The session will involve a 10-min duration of transcranial direct current stimulation (tDCS) priming, followed by a 192-s period of transcranial magnetic stimulation (TMS) test. Transcranial stimulation will be specifically targeted toward the left primary motor cortex. Measurements of motor evoked potentials will be taken at several time points: baseline, after tDCS, and after TMS. Coma Recovery Scale-Revised will be conducted both baseline and after TMS.

**Discussion:**

Studying whether homeostatic plasticity is preserved in patients with DoC is beneficial for gaining a better understanding of their brain condition. If the homeostatic plasticity of patients with DoC is impaired, then NIN, which are based on altering synaptic plasticity in healthy individuals to achieve stimulating effects, may not be directly translatable to the therapeutic interventions for patients with DoC. Instead, the homeostatic plasticity of patients should be restored before implementing the intervention.

## 1 Introduction

Consciousness, which consists of arousal and awareness, is the subjective projection of the human brain onto the objective world. When the brain is subjected to ischemia, hypoxia, external impact and other brain damage, there may be a loss of consciousness, and severe brain damage can even lead to disorders of consciousness (DoC) (1). Following brain injury, some patients may enter a state of coma characterized by a lack of both arousal and awareness (2). Unresponsive wakefulness syndrome (UWS) (3) is diagnosed when the patient recovers from a coma and reappears the sleep–wake cycle, but only shows reflexive movements and still has no awareness. Some patients may further transition to minimally conscious state (MCS), during which they exhibit some intentional behaviors. This manifestation of consciousness is fluctuating yet replicable (4). When patients improve further on the basis of MCS, they are thought to no longer have consciousness disorders, but mainly motor and cognitive impairment.

As an extremely disabled group, DoC patients impose a significant burden on their families and society. To address this challenge, various treatments have been proposed for these patients, with non-invasive neuromodulation (NIN) technology, such as transcranial direct current stimulation (tDCS) and transcranial magnetic stimulation (TMS), emerging as a standout option. These methods are portable, bedside-friendly, and capable of modulating brain activity. However, their efficacy remains inconsistent. While some studies demonstrate positive outcomes (5, 6), a recent trials have questioned the effectiveness of tDCS (7). Moreover, many NIN interventions produce mixed results, possibly due to the failure to account for the impact of the pre-treatment brain state, such as homeostatic plasticity, on the efficacy of NIN (8).

Although the mechanisms underlying NIN remain unclear, current research indicates that these stimuli affect neuronal activity by influencing synaptic plasticity, thereby adjusting the excitability of the cerebral cortex (9). Synaptic plasticity can be further categorized into Hebbian plasticity with positive feedback and homeostatic plasticity with negative feedback (10). Hebbian plasticity can specifically modify synapses to enhance [Long-term Potentiation (LTP)] or weaken [Long-term Depression (LTD)] synaptic transmission (11), a process thought to underlie the neurophysiology of learning and memory (12). However, this positive feedback mechanism can result in over-excitation or over-inhibition in the human body (13). Hence, a balancing homeostatic mechanism in the human body regulates Hebbian plasticity by stabilizing neuronal activity. This homeostatic plasticity counteracts the destabilizing effects of Hebbian plasticity, keeping neural activity within a physiologically significant range (14, 15).

Homeostatic plasticity can happen through two mechanisms: the sliding threshold theory (16) and the synaptic scaling theory (17). As per the sliding threshold theory, synaptic activity can modify the connection between synaptic input and neuron firing by adjusting a sliding threshold. Lower postsynaptic activity decreases the threshold for triggering LTP, increasing the likelihood of generating excitatory responses in synapses. In contrast, higher postsynaptic activity raises the threshold for LTP induction, encouraging the production of inhibitory responses. The synaptic scaling theory regulates neuronal activity by directly adjusting synaptic strength, either up or down. In a long-term LTD-like environment, excitatory synapses and Na ion channels increase, facilitating neuron excitation. Conversely, in a long-term LTP-like environment, inhibitory synapses and K ion channels increase, hindering excitatory responses. These mechanisms help maintain the overall activity of the neuronal network within a suitable range, preventing excessive excitation or inhibition.

Given the negative feedback mechanism of homeostatic plasticity, we can propose a priming-test preconditioning TMS tool that is more effective than the current stimulation alone. Initially, subjects will receive inhibitory priming to trigger their homeostasis response, followed by an excitatory test. As a result of the preceding inhibitory treatment, the neural network near the subject’s target area will more readily generate an excitatory response, leading to a more significant neural network activity during the excitatory test compared to the excitatory stimulus alone. Homeostatic plasticity plays a critical role in maintaining neural stability; thus, its impairment could limit the efficacy of NIN interventions. Research on myasthenia gravis (18), dystonia (19–21), Parkinson’s disease (22), Alzheimer’s disease (23), depression (24) and other psychiatric disorders (25) have highlighted deficiencies in homeostatic plasticity among patients, raising concerns about its preservation in DoC patients.

Therefore, prior to administering the combined stimulation to DoC patients, it is crucial to investigate the preservation of homeostatic plasticity in these individuals. This study is guided by two hypotheses: firstly, that DoC patients exhibit impaired homeostatic plasticity, with the degree of impairment correlated with their level of consciousness; and secondly, that patients who retain homeostatic plasticity may have a more favorable prognosis. To explore the retention of homeostatic plasticity in DoC patients, this study will employ two NIN technologies, tDCS and TMS, as assessment tools.

## 2 Methods

### 2.1 Design

We design a prospective, randomized, double-blind, repeated-measures crossover experiment to explore the preservation of homeostatic plasticity in patients with DoC. This study is registered in the Chinese Clinical Trial Registry (ChiCTR2300077784^1^), and protocol is aligned with the Declaration of Helsinki, approved by the Science Research Ethics Committee at the School of Basic Medicine, Hangzhou Normal University (20231024).

### 2.2 Participants

This study will include 15 patients with MCS, 15 patients with UWS, and 15 healthy subjects sourced from various hospitals. Before enrollment, each DoC patient will undergo five assessments using the Coma Recovery Scale-Revised (CRS-R) over a 10-day period conducted by assessment experts. The best result from the five assessments will be considered as the patient’s current level of consciousness, and assessment experts will assess if the patient meets the inclusion criteria during the evaluation process.

The criteria for inclusion and exclusion are outlined in Table 1.

**Table 1:** Inclusion and exclusion criteria.

Inclusion criteria	Exclusion criteria
Diagnosed in DoC with 5 times CRS-R assessments;	Active seizures or a history of mental illness;
Disease onset more than 28 day;	Metal implants in the skull;
Intact skin and anatomical structure;	Lack of motor evoked potential from right first dorsal interosseous muscle;
Right-handed as per Edinburgh Handedness Inventory;	Unstable vital signs;
Stable central nervous system drug therapy for 1 week before enrollment.	Use sedatives, sodium or calcium channel blockers.

DoC, disorders of consciousness; CRS-R, Coma Recovery Scale-Revised.

### 2.3 Sample size

The sample size calculations had been performed using G*power 3.1 (26) with prior power analysis. Based on previous studies of DoC patients (27), the effect size f had been calculated to be 0.3. A three-way repeated measures ANOVA had been conducted with test power (1-*β*) set at 80% and the type I error rate (*α*) of 5%. Taking into account an allocation ratio of 1:1:1 and a 20% data loss rate, this had resulted in a minimum of 15 participants per group.

### 2.4 Procedures

The study process will begin as early as 28 days after the patient’s injury, and the entire experimental process will be finished within 10 days. After informing the patient’s legal representative about the experimental content and potential adverse reactions, written informed consent will be obtained from them. They will be advised that they can choose to exit the study at any time without the requirement for providing a reason. After informing the healthy subjects of the experimental content and potential adverse reactions, they will sign the informed consent form.

**Figure 1:**
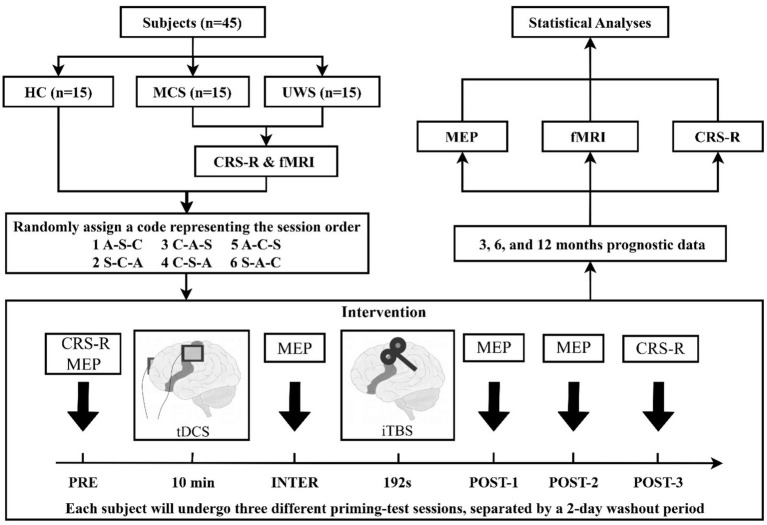
Study flow diagram. HC, health controls; MCS, minimally conscious state; UWS, unresponsive wakefulness syndrome; CRS-R, Coma Recovery Scale-Revised; fMRI, functional magnetic resonance imaging; EEG, electroencephalography; MEP, motor evoked potential; tDCS, transcranial direct current stimulation; iTBS, intermittent theta burst stimulation; A, anodal tDCS; C, cathodal tDCS; S, sham tDCS.

Before the trial, DoC patients will have the option to undergo a functional magnetic resonance imaging (fMRI) scan, and they can choose to decline. Over a period of 10 days, five CRS-R assessments, and an fMRI scan will be completed. The study protocol is a repeated-measures crossover design. According to different tDCS priming protocols (anode, cathode, sham), the trial will be divided into three distinct sessions. Each session will involve 10 min of tDCS priming followed by 192 s of TMS test, with both transcranial stimulations specifically targeted toward the left M1. We will measure the motor evoked potential (MEP) amplitude at four time points: baseline (PRE), after tDCS (INTER), and at 5 and 15 min following TMS (POST-1, POST-2). CRS-R data will be collected at baseline (PRE) and 20 min after TMS (POST-3). Upon enrollment, participants will receive a serial number based on their order of enrollment, with each serial number corresponding to a randomly generated session order. Subjects will then undergo three sessions following the designated session order, with a two-day washout period between each session. The entire trial will be completed in 5 days. Patient prognostic data will be gathered at 3, 6, and 12 months after the conclusion of the study. Figure 1 provides a detailed illustration of the study flow.

Adverse events will be promptly recorded and reported during the trial. Following this, experts will evaluate the connection between adverse events and the trial based on previous literature reports. If a severe adverse event is determined to be linked to the intervention, the trial will be immediately halted, and necessary medical actions will be taken for the patient until the concern are properly resolved.

### 2.5 Transcranial direct current stimulation

Homeostatic plasticity will be examined through a priming-test approach, where priming stimuli will induce and test stimuli will capture the homeostatic response (14). Previous research (28) has suggested that the fidelity of homeostatic response occurs when the prime protocol does not lead to noticeable changes in basal synaptic transmission. And prior studies (29) in mice models have demonstrated that anodal tDCS-induced LTP does not impact the efficiency of basal synaptic transmission. Therefore, tDCS is deemed the most suitable priming method, this study will also employ tDCS to trigger a homeostatic response. Given that the regulation of homeostatic plasticity involved both upward (30) and downward (31) homeostasis, anodal and cathodal tDCS were going to be used to prime upward and downward homeostasis, respectively. Sham tDCS was intended to serve as a control to evaluate the success of the priming process.

Continuous tDCS will be conducted using a DC stimulator. A steady 1 mA current will be delivered via wet sponge electrodes (7 cm × 5 cm) placed on the left M1 region and the right contralateral supraorbital area. The tDCS polarity will be determined by the electrode on the left M1. In the case of sham tDCS, only a small current pulse will occur every 550 ms (110 μA over 15 ms) instead of the actual stimulation current. The peak current will last for 3 ms, creating a brief skin sensation similar to real tDCS but lacking therapeutic benefits. To ensure the double-blind procedure, the real and sham stimulation will be managed by the code linked to the DC stimulator. Before each stimulation session, the researcher will receive the code of the session (unaware of the type of stimulation), and will start the stimulation by inputting the code to activate the DC stimulator.

### 2.6 Transcranial magnetic stimulation

Due to the specificity of these patients, intermittent theta burst stimulation (iTBS) will be utilized in this study to prevent fatigue from prolonged testing. Previous research (32) has proposed that iTBS, as opposed to conventional repetitive TMS, can expedite changes in neural activity while maintaining clinical effectiveness.

Both single-pulse TMS and iTBS will employ a rapid stimulator and a standard figure-of-eight coil. The coil will be placed tangentially on the scalp, with the handle angled 45° from the midline. TMS will be consistently applied over the optimal site (hotspot) to evoke the largest MEP in the relaxed right first dorsal interosseus (FDI) muscle. The individual resting motor threshold (RMT) will be evaluated, and the effects following transcranial stimulation will be explored using single-pulse TMS. RMT will be defined (33) as the minimal stimulation intensity required to generate a TMS-induced MEP peak-to-peak amplitude of ≥50 μV in at least half of the 10 consecutive trials. The iTBS pattern will comprise sets of bursts of 3 pulses at 50 Hz repeated at 5 Hz; a 2 s train of TBS will be delivered every 10 s for a total of 192 s (600 pulses). The stimulation intensity will be set at 90% of the individual RMT.

### 2.7 Cortical excitability measurements

This study will evaluate the cortical excitability of the motor cortex at four time points: BASELINE, INTER, POST1, and POST2, to investigate if priming stimuli induce and test stimuli capture the homeostatic response. Single-pulse TMS will be utilized to stimulate the motor cortex and generate MEPs in the peripheral muscles. The amplitude of these MEPs will indicate motor cortical excitability. We will administer 10 single-pulse TMS (stimulation intensity: 120%RMT; time interval: 5 s) to target the left M1 at four time points. Motor cortex excitability will be represented by averaging the peak-to-peak amplitudes of MEPs induced on the FDI muscle of the right hand, recorded during the experiment. The FDI muscle will be chosen for its small, localized, and palpable nature (34). MEPs will be recorded using the Ag-AgCl surface electrode provided with the TMS device. The EMG signal will undergo filtering and digitization using an analog–digital converter, then will be processed with low-pass, high-pass, and 50 Hz-Notch-Filter before being storage in a personal computer for offline analysis.

### 2.8 Behavioral assessments

The level of consciousness of DoC patients will be evaluated using the CRS-R, which consists of six subscales with a total of 23 items (35). The patients’ consciousness level will be evaluated across six dimensions: auditory, visual, motor, oromotor/verbal, communication and arousal. Comparing the total score, the best score of each subscale can more accurately reflect the patient’s condition. Due to the fluctuating consciousness levels in these patients, we will select the best of five assessments over a 10-day period to represent the patient’s baseline consciousness level. In each session, considering the limited duration of post-effects induced by transcranial stimulation, CRS-R evaluations will be conducted before and after stimulation, focusing on specific items, including the best items from each subscale at baseline and those that are one level above or below the best items. The scores of these specific items will be used to assess whether each session had a behavioral impact on the patient’s consciousness. Patients will undergo reassessment using CRS-R at 3, 6, and 12 months after the conclusion of the trial to collect prognostic data.

It’s worth noting that, in addition to DoC, there are various conditions associated with impaired levels of consciousness. The clinical symptoms of these conditions are summarized in Table 2.

**Table 2:** Symptoms associated with consciousness states.

State	Consciousness level	Clinical features and manifestations
Alertness/Wakefulness	Full	Full environmental attention; Normal conversation and behavior; Intact orientation; Appropriate responses to all stimuli
Somnolence	Mild impairment	Decreased attention; Slowed responses; Easily arousable; Normal response to strong stimuli
Lethargy	Moderate impairment	Delayed responses; Slurred speech; Requires repeated stimulation; Limited environmental interaction
Stupor	Severe impairment	Responds only to vigorous stimulation; Unable to maintain wakefulness; Minimal verbal response; Limited to pain responses
Coma	Complete loss	Unarousable; No spontaneous behavior; Only reflexive responses; No sleep–wake cycle
Akinetic Mutism	Partially preserved	Eyes open with preserved wakefulness; No spontaneous speech/movement; Limited environmental response; Preserved arousal mechanisms
Apallic Syndrome	Extremely severe impairment	Decorticate posturing; Preserved sleep–wake cycles; No purposeful behavior; Primitive reflexes only
Unresponsive Wakefulness Syndrome	Extremely severe impairment	Preserved sleep–wake cycle; No purposeful behavior; Reflexive responses only; No cognitive function
Minimally Conscious State	Severely impaired with partial preservation	Intermittent purposeful behavior; Basic command following; Inconsistent but reproducible responses
Locked-in Syndrome	Fully preserved	Preserved consciousness; Complete paralysis except eye movement; Intact cognition; Communication through eye movements
Delirium	Fluctuating impairment	Fluctuating attention and awareness; Disorientation; Perceptual disturbances; Unstable behavioral patterns
Catatonia	Variable	Abnormal motor behavior; Fixed posturing; Variable responsiveness; Possible excitement or stupor

### 2.9 fMRI scan

Before commencing the experiment, T1 scans will be conducted to confirm the integrity of patients’ M1 are intact and that the motor cortex excitability can be detected using TMS-MEP. Resting state fMRI data will be gathered post-experiment for further analysis to explore neuroimaging differences between patients with and without homeostatic plasticity.

### 2.10 Data safety and management

In addition to collecting behavioral, electrophysiological, and neuroimaging data, the researchers will also gather data from the case report forms (CRF). The study will store all electronic data in the Institute’s database and will securely store paper data in the Institute’s lockers. The identity of the subjects involved will be concealed when the above data is used for publication.

### 2.11 Statistical analysis

A single transcranial stimulation typically leads to limited behavioral improvement but increases cortical excitability due to its immediate effects (36). Therefore, the primary focus of this study will be to analyze the trend of MEP changes over time in different priming types, comparing healthy individuals with DoC patients. At the individual level, the cathodal tDCS group will be compared with the sham group. The primary outcome of this study will be MEP, a measure used to assess neurophysiological changes. Since the baseline MEP in each session will be influenced by various factors such as the individual’s daily state, MEP data from different sessions will lack comparability. Therefore, all MEP data will be normalized in this study (normalized MEP = MEP/MEP baseline). The normalized MEP data will be subjected to an analysis of variance (ANOVA) to explore the retention of homeostatic plasticity in DoC patients and to determine whether the combination of cathodal tDCS and iTBS is superior to iTBS alone.

While a single stimulus may not directly lead to a change in behavior, this study will conduct a CRS-R assessment (specific item) before and after each session to ensure that any potential improvements are not overlooked. Homeostatic plasticity, as a fundamental characteristic of synapses, can impact a range of synaptic activities and may even influence levels of consciousness and prognosis. Therefore, the CRS-R score, which assesses the level of consciousness, will be considered as a secondary outcome of this study.

Patients will be categorized into subgroups based on consciousness levels and prognosis. For consciousness levels, patients will be classified into the MCS or UWS group based on the best result from five baseline CRS-R assessments, To classify a CRS-R record as reflecting MCS, the participant must achieve at least one of the following criteria: a score of 1 on the communication subscale (intentional but non-functional yes/no communication), a score of 3 or higher on the auditory subscale (command-following), a score of 2 or higher on the visual subscale (visual fixation or better), a score of 3 or higher on the motor subscale (localization to noxious stimulation or better), or a score of 3 on the oromotor/verbal subscale (intelligible verbalization). If none of these criteria are met, the CRS-R record will be classified as reflecting UWS (37). For prognosis, patients will be evaluated at 3, 6, and 12 months, with those showing an increase of over 11 Rasch units (approximately 4–6 CRS-R points) (38) classified as the good prognosis group, while others will be placed in the poor prognosis group. A preserved homeostatic plasticity response is defined as a significant modulation of MEP amplitude following the priming-test protocol, consistent with the sliding threshold theory of synaptic plasticity. Its preservation was further compared between the MCS and UWS groups, as well as between the good and poor prognosis groups.

In this study, statistical analysis will be conducted using IBM SPSS Statistics 26 software. The collected data will first undergo normal distribution testing via Kolmogorov–Smirnov tests. If the data meets the criteria for a normal distribution, a three-way repeated measures ANOVA will then be carried out. However, if the data does not adhere to a normal distribution, Friedman’s ANOVA will be utilized instead. Following a significant outcome from the ANOVA, two-way ANOVAs will be conducted within each group to explore temporal changes in mean MEP amplitude. Additionally, Pearson correlation will be used to examine the relationship between homeostatic plasticity retention, consciousness, and prognosis. A significance level of *p* < 0.05 will be employed for determining statistical significance.

## 3 Discussion

The uniqueness of human consciousness arises from its dynamic interaction with complex communication, tool use, and social behavior. As an irreducible emergent phenomenon, consciousness is the core element that grants individuals moral status, which is particularly critical in ethical decision-making for DoC patients. Since some DoC patients exist in a borderline state between consciousness and unconsciousness, establishing an accurate classification system for unconscious states has become an urgent need in clinical practice (39, 40).

Non-consciousness refers to neurophysiological processes entirely detached from the psychological domain. This state is typically observed in some UWS patients. These patients exhibit brain activity lasting less than 100 ms, characterized by pure stimulus–response patterns without the foundation for self-awareness. Thus, determining their moral status requires careful consideration. Another group of UWS patients exists in a state of unconsciousness, which refers to mental processes that cannot be actively perceived or controlled but profoundly influence motivation, emotion, behavior, and decision-making. Their brain activity lasts approximately 220 ms, suggesting the potential for primary information integration mechanisms, though lacking explicit expression of conscious experience. Subconsciousness represents subliminal mental content that can be conditionally activated, typically associated with MCS. Brain activity lasting over 300 ms reflects intermittent environmental awareness, providing a critical window for the recovery of consciousness (41).

This classification system not only enables precise differentiation of clinical subtypes but also guides prognosis evaluation and treatment strategy development. In this process, homeostatic plasticity has been identified as a key bridging mechanism. This neuroadaptive mechanism, present at micro (30, 31), meso (15, 42), and macro (43) levels, reflects levels of consciousness (27) while promoting spontaneous functional reorganization after brain injury. Studies have shown that homeostatic plasticity allows the brain to recover spontaneously after large-scale neuronal disturbances (43). Therefore, maintaining homeostatic plasticity may be closely associated with favorable outcomes in DoC patients.

In this study, tDCS and TMS will be used to detect homeostatic plasticity retention in patients with DoC. tDCS applies low-amplitude (1–2 mA) direct current to the brain via two scalp electrodes, modulating the threshold of action potential generation, thereby depolarizing (anode) or hyperpolarizing (cathode) individual neurons (9, 44, 45). TMS is a technique that integrates nerve stimulation and cortical excitability detection. It uses electromagnetic induction to induce secondary currents in subcortical neurons, thereby activating neurons to change physiological processes in the brain (46, 47).

While two NTBS technologies, tDCS and TMS, are widely used in the field of treatment for DoC, the selection of parameter and targets often relies on studies related to other diseases or healthy individuals. In DOC, the most common targets are the M1 or the dorsolateral prefrontal cortex (DLPFC) rather than the anterior cingulate cortex or posterior cingulate gyrus emphasized by many theories of consciousness. M1 is frequently targeted in the motor rehabilitation of stroke patients, while DLPFC is primarily employed in depression treatment (48). Such empirical transplantation carries significant theoretical risks—severe brain injury may fundamentally alter homeostatic plasticity mechanisms, and directly applying neuromodulation parameters from healthy populations may lead to diminished efficacy or even induce secondary damages such as epilepsy.

Based on these findings, we propose a stepwise intervention strategy. The primary task is to assess the retention of homeostatic plasticity in DoC patients. For patients with impaired homeostatic plasticity, priority should be given to restoring this mechanism before proceeding with neuromodulation treatments to prevent uncontrolled risks caused by impaired negative feedback regulation. Previous successes in dopamine modulation for Parkinson’s disease (22) suggest that the neurotransmitter system may serve as an important regulatory target. In some cases, impaired homeostatic plasticity might even indicate the irreversibility of a patient’s condition.

For patients with preserved homeostatic regulation capacity, we recommend a “suppression-excitation sequential stimulation” model to avoid the risks of overactivation. Previous studies have indicated that short-term NIN stimulation typically does not lead to immediate behavioral improvements; instead, it primarily induces changes in electrophysiological indicators (49–51). Therefore, repeated interventions are necessary for DoC patients. Future studies could investigate the effects of continuous preconditioning TMS over a duration of 4 weeks or longer, with pre- and post-intervention evaluations using CRS-R or multimodal assessments to assess the long-term effects of combined stimulation. For complications such as epilepsy, innovative applications of homeostatic plasticity principles could enable localized excitability regulation (52). For instance, excitatory cortical stimulation techniques could directly target hyperexcitable epileptic regions, reducing cortical excitability through a “fight fire with fire” approach. Additionally, standardized preprocessing (8, 53) could effectively mitigate the impact of individual heterogeneity on intervention outcomes.

This study aims to offer objective evidence to enhance our understanding of synaptic plasticity in the brains of DoC patients. Considering the significance of homeostatic plasticity, its retention status will significantly impact the clinical management of DoC patients and could potentially lead to fundamentally changes in current treatment protocols for this patient population.

## Footnotes

^1^ https://www.chictr.org.cn/showproj.html?proj=209755
